# A City Hospital for Atlanta

**Published:** 1888-08

**Authors:** 


### A CITY HOSPITAL FOR ATLANTA.

There is now a movement on foot in this city, which we hope will result in securing the benefits of a first-class hospital to the indigent sick of our community.

At the last meeting of the City Council the relief committee recommended withdrawing from the Ivy Street Hospital the appropriation formerly allowed it, on the ground that the city patients sent to this institution were not properly cared for. This recommendation was almost unanimously adopted. A committee of some of our most prominent physicians and lay citizens have been working for several months, elaborating plans for building a general city hospital, free from sectarian or private interests, and managed solely for the good of the people. This committee will appear before the City Council in the Fall and ask for an appropriation for this purpose. We wish them God-speed.

